# MSCs Deliver Hypoxia‐Treated Mitochondria Reprogramming Acinar Metabolism to Alleviate Severe Acute Pancreatitis Injury

**DOI:** 10.1002/advs.202207691

**Published:** 2023-07-06

**Authors:** Zhengyu Hu, Dongyan Wang, Jian Gong, Yan Li, Zhilong Ma, Tingyi Luo, Xuyang Jia, Yihai Shi, Zhenshun Song

**Affiliations:** ^1^ Department of Hepatopancreatobiliary Surgery Shanghai Fourth People's Hospital School of Medicine Tongji University Shanghai 200434 China; ^2^ Department of General Surgery Shanghai Tenth People's Hospital School of Medicine Tongji University Shanghai 200072 China; ^3^ Department of General Surgery The First Affiliated Hospital of Anhui Medical University Hefei Anhui Province 230032 China; ^4^ Department of Gastroenterology Shanghai Pudong New Area Gongli Hospital Shanghai 200135 China; ^5^ Department of Gastroenterology Shanghai Tenth People's Hospital School of Medicine Tongji University Shanghai 200072 China; ^6^ Department of Pancreatic Surgery Fudan University Shanghai Cancer Center Shanghai 200032 China

**Keywords:** cargocytes, mesenchymal stem cells, metabolic reprogramming, mitochondria transfer, severe acute pancreatitis

## Abstract

Mitochondrial function impairment due to abnormal opening of the mitochondrial permeability transition pore (MPTP) is considered the central event in acute pancreatitis; however, therapeutic choices for this condition remain controversial. Mesenchymal stem cells (MSCs) are a family member of stem cells with immunomodulatory and anti‐inflammatory capabilities that can mitigate damage in experimental pancreatitis. Here, it is shown that MSCs deliver hypoxia‐treated functional mitochondria to damaged pancreatic acinar cells (PACs) via extracellular vesicles (EVs), which reverse the metabolic function of PACs, maintain ATP supply, and exhibit an excellent injury‐inhibiting effect. Mechanistically, hypoxia inhibits superoxide accumulation in the mitochondria of MSCs and upregulates the membrane potential, which is internalized into PACs via EVs, thus, remodeling the metabolic state. In addition, cargocytes constructed via stem cell denucleation as mitochondrial vectors are shown to exert similar therapeutic effects to MSCs. These findings reveal an important mechanism underlying the role of mitochondria in MSC therapy and offer the possibility of applying mitochondrial therapy to patients with severe acute pancreatitis.

## Graphical abstract

MSCs deliver hypoxia‐treated functional mitochondria to pancreatic acinar cells (PACs) via extracellular vesicles (EVs), which reprogram the metabolic state of PACs, maintain ATP supply, and exhibit an excellent injury‐inhibiting effect. In addition, Cargocytes constructed via stem cell denucleation as mitochondrial carriers show excellent therapeutic effects in vivo.

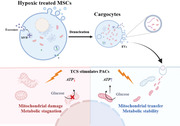

## 1 Introduction

Acute severe pancreatitis (SAP) is a common and fatal inflammatory disease of the exocrine pancreas, but, lacks specific and effective treatment.^[^ ^1^ ^]^ SAP is associated with gallstone disease, smoking, alcohol consumption, diabetes, obesity, and hyperlipidemia. The incidence of SAP is increasing annually and can seriously aggravate the economic burden on the public health system.^[^ ^2^ ^]^ Almost 30% of SAP patients will suffer from chronic pancreatitis due to pancreatic damage after the acute phase.^[^ ^3^ ^]^ Thus, there is an urgent need for a dedicated SAP treatment to promote survival, alleviate pancreatic damage and reduce the incidence of in intensive care.

Although the pathogenesis of SAP has yet to be fully elucidated, the abnormal opening of the mitochondrial permeability transition pore (MPTP) due to intracellular calcium overload induced by toxic substances (bile acids, ethanol and its metabolites) can result in the loss of transmembrane potential necessary for adenosine triphosphate (ATP) synthesis; this is thought to be a key event in the development of SAP.^[^ ^4^ ^]^ Diminished ATP production induces defective autophagy, disordered endolysosomal trafficking, building up activated digestive enzymes, and further driving intracellular and extracellular injury.^[^ ^4^, ^5^ ^]^ Pancreatic acinar cells (PACs), the initial cell population involved in SAP injury, are typically non‐excitable exocrine cells with a high secretory turnover and are heavily dependent on mitochondria for ATP production.^[^ ^6^ ^]^ Therefore, protecting the mitochondrial function of PACs, and the reprogramming cellular metabolism, is considered as a potential therapeutic strategy.

Mesenchymal stem cells (MSCs) are an important member of the stem cell family and have been used extensively to treat various inflammatory diseases in animal models and humans due to their strong immunomodulatory and anti‐inflammatory abilities.^[^ ^7^ ^]^ Previously, our group elucidated some of the important mechanisms of MSCs in the treatment of SAP.^[^ ^8^ ^]^ However, because of the rapid progression of SAP and the potential oncogenic risk of MSCs,^[^ ^9^ ^]^ clinical trials relating to the use of MSCs for SAP have yet to be approved. In recent years, numerous studies have shown that soluble factors and extracellular vesicles (EVs) (exosomes and microvesicles) secreted by MSCs can be used as an alternative option to suppress inflammation and facilitate tissue repair.^[^ ^10^ ^]^ As research progressed, these EVs were proven to contain not only proteins, mRNAs, mi‐RNAs, they also contained some other cellular components (e.g., mitochondria).^[^ ^11^ ^]^ Deciphering the key therapeutic substances is a crucial issue and current research aims to promote clinical trials relating to cell‐free delivery vehicles (CFDV) for SAP.

Over the past decade, numerous studies have shown that mitochondria from healthy MSCs can be transferred to damaged cells and are an effective method of regeneration.^[^ ^12^ ^]^ MSC mitochondria can be transferred to damaged cells through Miro 1‐mediated tunnel tube formation,^[^ ^13^ ^]^ microvesicle formation, gap junctions or cell fusion. However, the complete mechanism underlying induced mitochondrial transfer has not been elucidated, and the role of transferred mitochondria in the host cell remains unclear.

In this study, we pretreated human umbilical cord‐derived MSCs (hUC‐MSCs) under hypoxic conditions (5% O_2_) and found that they exhibited strong anti‐necrotic and anti‐inflammatory effects against experimental SAP by donating their “functional mitochondria.” We found that the transfer of mitochondria to the PACs can alleviated the stress caused by bile acids, maintain the mitochondrial membrane potential, and ensure a relatively stable metabolism and ATP supply, thereby preventing the “waterfall effect” amplified by the accumulation of pro‐inflammatory factors in SAP. Importantly, we designed an enucleated MSCs that delivers mitochondria to the site of pancreatitis injury, exerting both anti‐damage and anti‐inflammatory effects. This strategy also makes it possible to use MSC products in clinical trials to treat acute inflammatory diseases. Our study demonstrates that hypoxia‐pretreated mitochondria can be taken up by PACs, making them more resistant to toxin stimuli, to maintain a relatively stable metabolic environment and energy supply, and thus, combat inflammatory damage.

## 2 Results

### 2.1 Hypo‐MSCs Were More Effective Than Norm‐MSCs in the Treatment of TCS‐Induced SAP

**Figure 1:**
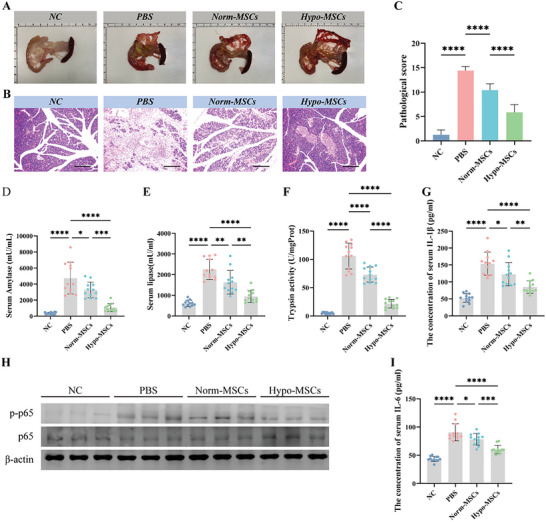
Hypoxic pretreatment of UC‐MSC enhances their therapeutic efficacy in SAP. A) Representative photos of pancreatic tissue after normoxia or hypoxia treated MSC for SAP. B) Pancreas slices stained with Hemotoxylin and eosin (H&E) from different groups on days 3, scale bar: 200 µm. C) Pathological scores for H&E staining in the pancreas. Concentration of D) amylase and E) lipase in the serum. F) The level of trypsin activity in the pancreas. Concentration of G) IL‐1*β* and H) Pancreatic levels of phosphorylated and total p65 (NF‐*κ*B) were measured by Western blotting (WB). Each lane on WB represents an individual animal. I) IL‐6 in the serum.*p < 0.05, **p < 0.01, ***p < 0.001, and ****p < 0.0001.

Previous studies have demonstrated that hypoxic treatment can enhance the therapeutic effect of MSCs on inflammatory diseases and ischemic injuries.^[^ ^14^ ^]^ Therefore, in this study, we used hUC‐MSCs cultured under normal oxygen conditions (21% O_2_, Norm‐MSCs) and hypoxia‐treated MSCs (5% O_2_, Hypo‐MSCs) to treat Sodium Taurocholate (TCS)‐induced SAP in rats. We found that Hypo‐MSCs were more effective in alleviating inflammatory injury, with significantly fewer foci of necrotic calcification in pancreatic tissue (**Figure** **1**A). HE staining and pathology scoring were used to quantify the degree of injury (Figure 1B,C). Hypo‐MSCs also effectively reduced the serum levels of amylase and lipase (Figure 1D,E), inhibited the abnormal activation of trypsin in the pancreas (Figure 1F). In addition, Hypo‐MSCs reduced serum levels of IL‐1*β* (Figure 1G), IL‐6 (Figure 1I), and inhibited the activation of Nuclear Factor Kappa B (NF‐*κ*B, Figure 1H), which mediates AP inflammation. These substances, which reflect reduced levels of inflammation in the peripheral circulation, suggest that Hypo‐MSCs are indeed effective in controlling the progression of SAP.

### 2.2 MSCs Produces Therapeutic Effects in SAP Rats via Paracrine Secretion

As found in our previous study,^[^ ^15^ ^]^ neither Norm‐MSCs nor Hypo‐MSCs were substantially enriched into the damaged pancreatic tissue after tail vein injection. Most of the fluorescent signals remained in the liver or spleen (Figure S2, Supporting Information). This also suggests that the main therapeutic effects produced were caused by the effect of UC‐MSCs on the paracrine pathway (exosomes or EVs).

### 2.3 Mitochondria Play a Critical Role in the Treatment of Hypo‐MSC‐EVs

To investigate whether MSCs produce a critical therapeutic effect by transferring mitochondria via EVs to damaged rPACs, we collected CM from different treatments and isolated EVs by differential centrifugation. WB showed that the extracted EVs were positive for exosome markers (TSG101, CD63, CD9, and CD81) and negative for glucose regulatory protein 94 (Grp94), thus confirming that the extracted EVs were not contaminated with endoplasmic reticulum‐derived vesicles (Figure S3B, Supporting Information). The samples consisted of multiple particle clusters between 100 and 1000 nm in diameter, with diameters of 100–400 nm representing exosomes and small EVs, and sizes above 500 nm corresponding to larger EVs (Figure S3C, Supporting Information). While using TEM to observe the morphology of EVs (Figure S3D, Supporting Information), we noticed the presence of mitochondrial structure in some large vesicles (Figure S4A, Supporting Information). WB results also detected mitochondrial membrane marker (TOMM20) proteins in EVs (Figure S4B, Supporting Information). Further characterization using flow cytometry revealed that mitochondrial positive signals were detected in 5–15% of EVs isolated from MitoTrack Red pre‐stained MSCs (Figure S4C, Supporting Information).

**Figure 2:**
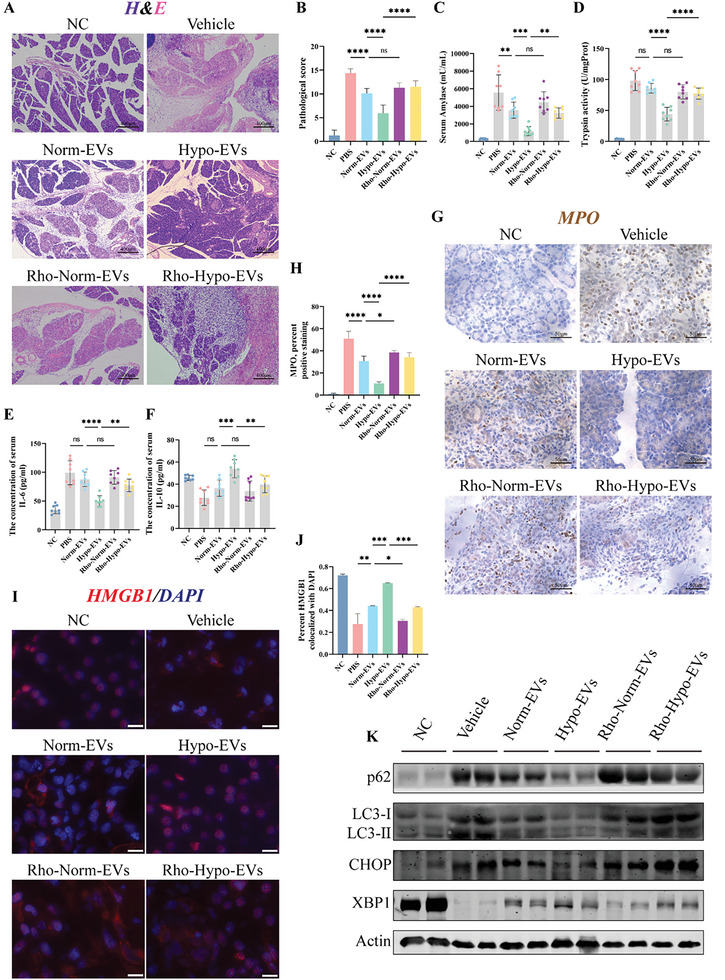
Mitochondria play a key role in the treatment of SAP with hypo‐MSC‐EVs. A) H&E stained of pancreas slices in different treatment groups, scale bar: 400 µm. B) Pathological scores for H&E stained in the pancreas. Concentration of C) amylase, E) IL‐6, and F) IL‐10 in the serum. D) The level of trypsin activity in the pancreas. G,H) Immunostained of pancreatic tissue for the neutrophil marker myeloperoxidase (MPO) to measure the degree of inflammatory infiltration in the scored pancreas. I,J) Subcellular localization of immunofluorescently labelled HMGB1 in pancreatic alveolar cells and statistical analysis using “coloc2,” scale bar: 50 µm. K) Markers/mediators in endoplasmic reticulum (ER) stress and autophagy pathways in pancreatic tissue were analyzed by WB. *p < 0.05, **p < 0.01, ***p < 0.001, and ****p < 0.0001.

Similar results to MSCs were found using different EVs for treatment, with Hypo‐EVs showing a better treatment effect (**Figure** **2**A–F). The therapeutic effect of EVs was significantly diminished after the inhibition of mitochondrial function with rhodamine 6G. Pancreatic tissue treated with Hypo‐EVs exhibited less neutrophil infiltration (Figure 2G,H) and cytoplasmic release of HMGB1(Figure 2I,J), a prototype of a damage‐associated molecular pattern (DAMP) released by injured cells. WB also demonstrated that treatment with Hypo‐EVs improved impaired autophagy and endoplasmic reticulum stress in SAP (Figure 2K). All indicators suggest that hypo‐EVs can inhibit SAP injury; however, these therapeutic effects were partially inhibited or disappeared in rhodamine 6G‐treated EVs. This suggests a critical role for mitochondrial function in MSC‐EVs for the treatment of SAP.

### 2.4 Differentially Expressed Genes between Hypo‐MSCs and Norm‐MSCs

**Figure 3:**
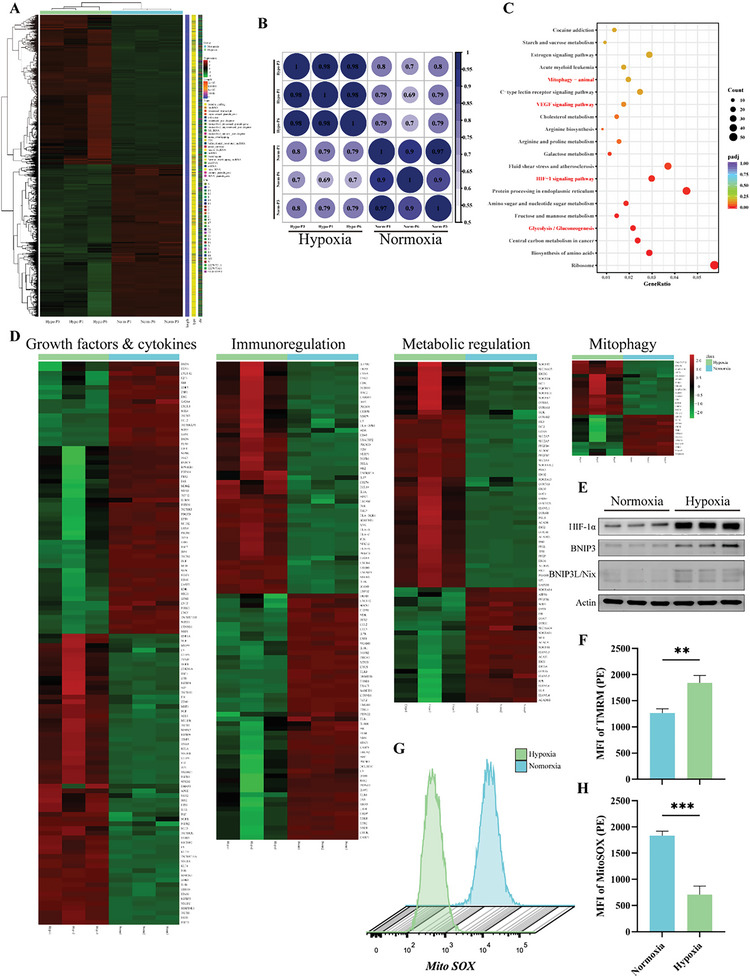
Hypo‐MSCs and Norm‐MSCs exhibit different gene expression profile by RNA‐seq analysis. A) Heat map indicated the difference in global gene expression profiles between Hypo‐MSCs (*n* = 3) versus Norm‐MSCs (*n* = 3). B) Pearson's correlation analysis demonstrated correlation of expression profiles between Hypo‐MSCs and Norm‐MSCs between P1–P6 generations. C) Kyoto Encyclopedia of Genes and Genomes (KEGG) enrichment analysis demonstrated pathway enrichment of difference‐expressed genes between Hypo‐MSCs and Norm‐MSCs. D) Heat map illustrated differential genes in cytokines, immune regulation, metabolism, and mitophagy in Hypo‐MSCs and Norm‐MSCs. E) Mitophagy‐related proteins expression in Norm‐MSCs and Hypo‐MSCs were analyzed by WB. HIF‐1*α*, Hypoxia Inducible Factor 1 Subunit Alpha; BNIP3, BCL2/Adenovirus E1B 19 kDa Protein‐Interacting Protein 3. F) Measured the mitochondrial membrane potential of Norm‐MSCs and Hypo‐MSCs by flow cytometry. G,H) Flow cytometry detected and quantified the mean fluorescence intensity (MFI) of superoxide in mitochondria. *p < 0.05, **p < 0.01, ***p < 0.001, and ****p < 0.0001.

To understand the molecular mechanisms underlying the therapeutic effects of Hypo‐MSCs, RNA‐seq analysis was used to determine the differential expression of the transcriptome between Norm‐MSCs and Hypo‐MSCs. Hierarchical clustering (**Figure** **3**A) shows the differential genes identified between Hypo‐MSCs and Norm‐MSCs. Pearson correlation analysis (Figure 3B) revealed non‐significant differences in the MSC transcriptome between generations of serum substitution cultures. KEGG enrichment analysis revealed differential gene enrichment in the “VEGF signaling,” “Mitophagy,” and “glycolysis” pathways (Figure 3C). Then, we remapped differential genes to the “Growth factors & cytokines,” “Immunoregulation,” “Metabolic regulation,” and “Mitophagy” categories based on clustering heatmaps (Figure 3D). Consistent with previous findings,^[^ ^16^ ^]^ hypoxia‐induced MSCs promote BNIP3 transcription through HIF‐1*α* to trigger mitochondrial autophagy, thereby maintaining the stability of mitochondrial quantity and quality (Figure 3E). Flow cytometric analysis confirmed that the hypoxic treatment of MSCs reduces superoxide accumulation in mitochondria (Figure 3G,H) and elevates the levels of mitochondria membrane potential (Figure 3F).

### 2.5 hUC‐MSC‐EVs Reprogrammed the Metabolic State of Injured rPACs by Transferring Functional Mitochondria

**Figure 4:**
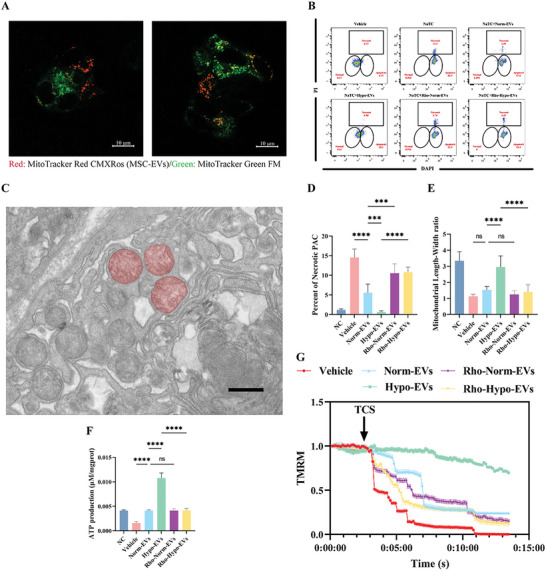
MSC‐EVs maintain morphology and structure of mitochondria in rat pancreatic acinar cells (rPACs) by transferring functional mitochondria. A) Representative live images of MSC‐EVs mitochondria internalisation in rPACs, scale bars: 10 µm. B) Representative flow cytometry dot blots for PI/Hochest33342 staining of TCS‐exposed rPACs grown alone or exposed to untreated EVs or Rho 6G‐treated EVs. Bottom‐left circle, % viable cells; bottom‐right circle, % apoptotic cells; upper‐square, % necrotic cells. C) TEM showing abnormally swollen mitochondria with absent ridges in the rPACs. D) Quantification of the necrosis percentages of rPACs exposed to TCS in different treatment groups. E) Quantification of mitochondrial length‐to‐width ratio. F) Quantification of ATP production in rPACs exposed to TCS in different treatment groups. G) Protection of Δ*ψ*m from TCS (5 mm) by pretreatment with untreated EVs or Rho 6G‐treated EVs. *p < 0.05, **p < 0.01, ***p < 0.001, and ****p < 0.0001.

Enhanced mitochondrial autophagy is thought to be a self‐protective mechanism for stem cells that respond to extreme environments by degrading damaged mitochondria. Therefore, we assume that hypoxic stem cells supply their own “superior” mitochondria to the rPACs to maintain mitochondrial function and metabolic homeostasis, thereby interrupting the vicious cycle of inflammatory amplification. To determine the mechanisms underlying the effect of mitochondrial transfer, we used rPACs to simulate SAP injury in vitro. Confocal microscopy showed that rPACs could easily capture MSC‐derived mitochondria in just 6 h (**Figure** **4**A). Internalized hypoxia‐treated mitochondria rPACs showed significantly less extensive necrosis upon stimulation with high concentrations of TCS (Figure 4B,D). Indeed, when exposed to high concentrations of TCS stimulation, PACs quickly exhibited mitochondrial dysfunction, with morphological swelling (Figure 4C,E; Figure S5, Supporting Information) accompanied by loss of membrane potential (Figure 4G). In contrast, rPACs loaded with hypoxic mitochondria produced more ATP (Figure 4F) in response to TCS stimulation; the membrane potential was maintained relatively stable (Figure 4G). Meanwhile, rhodamine‐6G inhibited these therapeutic effects, as shown in the in vivo experiments.

**Figure 5:**
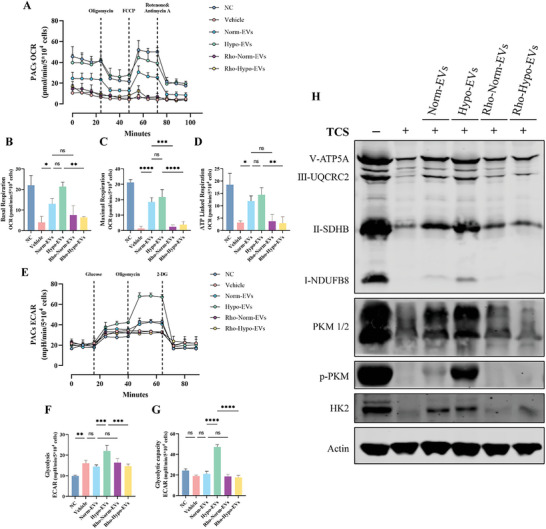
Hypo‐EVs transfer mitochondria enhance glycolytic flux and capacity of rat pancreatic acinar cells (rPACs). A) Assessed OCR in PACs cultured alone, TCS stimulated or stimulated after loading with different EVs. After the evaluation of basal respiration, oligomycin, FCCP, and rotenone/antimycin A were added as shown (*n* = 3). B) Basal respiration, C) maximal respiration, and D) ATP‐linked respiration of PACs cultured alone, TCS stimulated, or stimulated after loading with different EVs. E) Assessed ECAR in PACs cultured alone, TCS stimulated, or stimulated after loading with different EVs. After the evaluation of initial ECAR, glycose, oligomycin, and 2‐DG were added as shown (*n* = 3). F) Glycolysis, and G) Glycolytic capacity of PACs cultured alone, TCS stimulated, or stimulated after loading with different EVs. H) Markers/mediators in Oxidative phosphorylation (Oxphos) and glycolytic pathways in PACs were analyzed by WB. *p < 0.05, **p < 0.01, ***p < 0.001, and ****p < 0.0001.

In order to investigate the effect of MSC‐EVs on the metabolism of PACs, we measured the oxygen consumption rate (OCR) and the extracellular acidification rate (ECAR) of the PACs using the Seahorse assay. TCS significantly reduced basal, maximal, and ATP‐linked mitochondrial respiration in PACs and these effects were partially reversed by EVs, whereas rhodamine‐treated EVs were not (**Figure** **5**A–D). However, the difference in ATP‐linked respiration between Norm‐EVs and Hypo‐EVs was not significant, which contradicts with our measured ATP production (Figure 4F). Further measurements of the ECAR (Figure 5E) revealed that the calcium overload caused by TCS impaired the mitochondrial membrane potential and caused a compensatory increase in the glycolytic flux of PACs, while loading Hypo‐EVs not only upregulated the glycolytic flux (Figure 5F), but also increased the glycolytic reserve capacity of the PACs (Figure 5G).

WB also indicated that the metabolic environment of rPACs was almost in a collapsed state after TCS stimulation while the key enzymes of glycolysis and oxidative phosphorylation (OXPHOS) were reduced to varying degrees. In contrast, hypoxic mitochondria largely maintained the energy output of rPACs, as evidenced by the sustained levels of HK2, p‐PKM, ATP5A, SDHB (Figure 5H).

### 2.6 Reversal of Mitochondrial Function Drove the Metabolic Reprogramming of Pancreatic Tissue

To validate that hypoxic mitochondrion transfer caused a shift in pancreatic acinar metabolic pathways, we performed untargeted metabolomic analysis of pancreatic tissue from normal controls, untreated, and Hypo‐MSCs treated rats.

**Figure 6:**
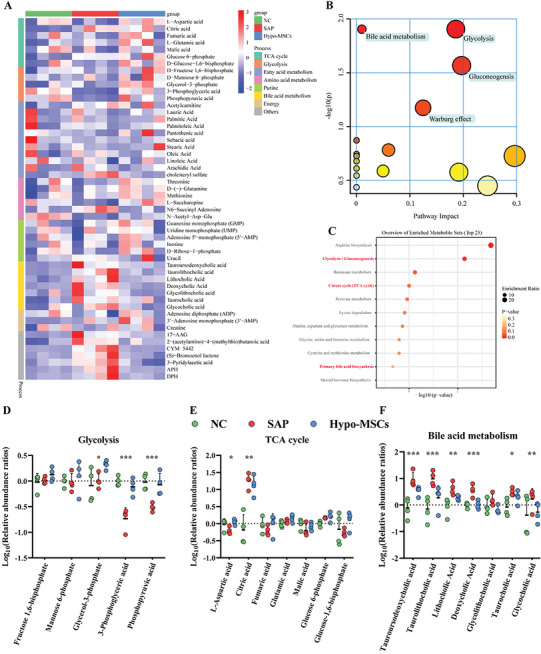
Reversal of mitochondrial function drives metabolic reprogramming in the pancreas. A) Unclustered heat map showing the relative abundance of 53 metabolites in the pancreas of NC, SAP, and Hypo‐MSCs treated groups. B) Integrative pathway and C) enrichment analysis using MetaboAnalyst of reversal metabolites in the pancreas after Cargocytes treatment. Dot plots of relative abundance of D) glycolysis‐associated metabolites, E) TCA‐associated metabolites, and F) bile acid metabolites in pancreas of NC or SAP or Cargocytes‐treated. *p < 0.05, **p < 0.01, ***p < 0.001, and ****p < 0.0001.

Based on the composition of the metabolites, principal component analysis (PCA) clearly distinguished between NC, SAP, and Hypo‐MSCs group (Figure S6A, Supporting Information). We constructed unsupervised hierarchical non‐clustered heat maps for the 53 metabolites identified in seven key energy‐producing metabolic pathways (**Figure** **6**A). Metabolites with reversed abundance before and after treatment were then selected for further analysis (Figure S6B,C, Supporting Information). Integrated pathway (Figure 6B) and enrichment analysis (Figure 6C) based on the KEGG database revealed that these metabolites were enriched in “glycolysis,” “TCA cycle,” and “bile acid metabolism.” SMPDB‐based enrichment analysis showed similar results and correlation coefficients were constructed between these differential metabolites (Figure S6D,E, Supporting Information). Comparison of the relative abundance of metabolites demonstrated that MSCs reversed the reduction of glycerol 3‐phosphate, 3‐phosphoglyceric acid, and phosphoenolpyruvate (Figure 6D) and the accumulation of citric acid (Figure 6E) in the damaged pancreas. These data partly reflect the results of in vitro experiments that hypoxia‐treated mitochondrial transfer drives metabolic changes in the damaged pancreas.

Moreover, the reduced abundance of bile acids indicated that pancreatic duct function was normal after MSC treatment (Figure 6F), thus allowing the smooth drainage of bile and pancreatic fluid into the intestine and preventing the aggravation of SAP due to obstruction by pancreatic duct injury.^[^ ^17^ ^]^

### 2.7 Cargocytes Carrying Hypoxic Mitochondria Produce Therapeutic Effects on SAP

**Figure 7:**
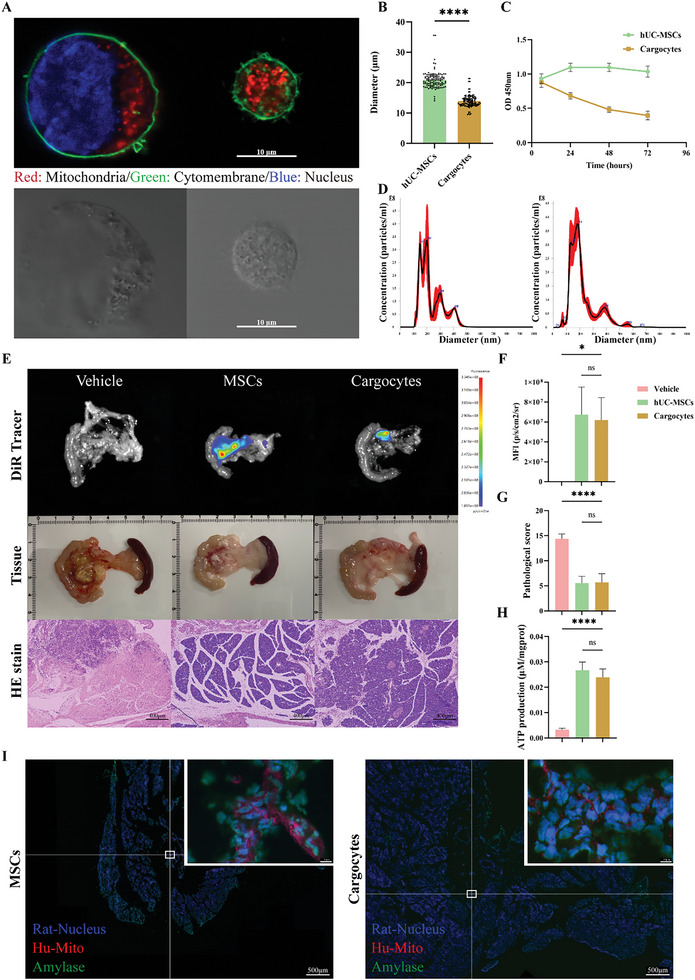
Cargocytes deliver hypoxia‐treated mitochondria to impaired rPACs to reverse mitochondrial function. A) Fluorescent image (upper) and brightfield image (down) show UC‐MSCs and Cargocytes in suspension. Scale bar, 10 µm. B) Average diameter of UC‐MSCs and Cargocytes in suspension. C) Cell viability of UC‐MSCs/Cargocytes in the adherent culture status over time. D) Size distribution of secreted EVs from UC‐MSCs (left panel) or Cargocytes (right panel). E) DiR in vivo tracer photos (top), pancreatic tissue photos (middle), and slices HE stain (bottom) show the effect of UC‐MSCs/Cargocytes on the inhibition of pancreatic injury. Scale bar, 400 µm. Quantification of F) the mean fluorescence intensity, G) pathological scores, and H) ATP production in the pancreas of vehicle, UC‐MSCs and Cargocytes groups. I) Rat pancreatic tissues were quickly frozen sectioned and stained with anti‐human mitochondrial antibody (red), amylase (green), and DAPI (blue). *p < 0.05, **p < 0.01, ***p < 0.001, and ****p < 0.0001.

Despite the key role of mitochondria, we did not observe a significant therapeutic effect in SAP rats when treated with enriched and purified mitochondria (Figure S7, Supporting Information). This could have been caused by several factors. For example, exposure to the high calcium environment of the serum may have caused swelling of the mitochondria or the inability of mitochondria to move to the injured cells. Another option is that isolated EVs could not be preserved in vitro for long periods of time. The prolonged storage of isolated EVs in vitro could also lead to mitochondrial dysfunction. Protecting mitochondria and delivering these organelles to the injured region for release should be a critical point in mitochondrial therapy. We were intrigued by the idea of Cargocytes, a technique that removes the nucleus of cells while retaining the organelles that produce energy and proteins.^[^ ^18^ ^]^ Cargocytes will not proliferate or implant permanently in a host, can sense induction by serum, and have a similar chemotactic ability to MSCs (Figure S8, Supporting Information). Following removal of the nucleus, the Cargocytes have a much smaller cell diameter (**Figure** **7**A,B) and remain relatively active in vitro for 72 h (Figure 7C; Figure S8A, Supporting Information). More importantly, we found that Cargocytes also secrete EVs and that the size distribution of Cargocytes‐EVs was similar to Hypo‐MSC‐EVs (Figure 7D; Figure S8D, Supporting Information). The use of Cargocytes to treat SAP rats resulted in similar therapeutic effects to Hypo‐MSCs (Figure 7E–H); IF confirmed that Cargocytes can deliver human‐derived mitochondria to rPACs (Figure 7I).

## 3 Discussion

Due to the instability in efficacy and potential oncogenic risk of MSCs, as well as the rapid changes in the course of SAP, clinical research on the treatment of SAP with MSCs has not been effectively carried out yet, despite the extensive work performed by our group in this area. Therefore, there is an urgent need to identify the critical substances in MSCs for the treatment of SAP to precisely and efficiently target injured tissues to reduce unnecessary side‐effects and improve efficacy. Experimental results revealed that MSCs exert a key therapeutic effect by delivering hypoxia‐treated mitochondria (Figures 2 and 4). EVs cannot be stored in vitro for long periods of time and large extractions are costly while no efficacy has been observed with purified mitochondrial (Figure S7, Supporting Information). Thus, we have constructed a unique CFDV‐based mitochondrial delivery method with reference to the previously established method of mammalian cell enucleation.^[^ ^19^ ^]^ Cargocytes have specific advantages over other CFDVs (i.e., exosomes, erythrocytes, or nano‐ghosts) in that: 1) they retain their energy and protein producing organelles (Figure 7A) and can actively migrate in response to specific chemokine gradients released from diseased tissue (Figure S8B, Supporting Information); 2) they lacking a rigid nucleus and have a smaller diameter (Figure 7A,B), thus, reducing the risk of possible vascular embolism during infusion, and do not proliferate in the host, and 3) Cargocytes have a lower cost of production and can be frozen and remain active in vitro as long as MSCs (Figure 7C; Figure S8, Supporting Information). These advantages suggest that cargo cells are a highly prospective clinical substitute for MSCs.

There is growing evidence that the impairment of mitochondrial function due to abnormal MPTP opening is a critical event in all studied types of acute pancreatitis.^[^ ^4^, ^20^ ^]^ The resulting amplification cascade of events, such as reduced ATP production, defective autophagy, zymogen activation, cytokine release, and the activation of phosphoglycerate metastable enzyme family member 5 (PGAM5), leads to an increasingly intense inflammatory response and ultimately to pancreatic necrosis. Most previous studies have focused their attention and therapeutic targets on inhibiting the abnormal opening of MPTP.^[^ ^21^ ^]^ Despite impressive results in animal studies, it is difficult to apply this treatment in the clinical setting. This is because preclinical models differ from human disease in that patients often already have severe abdominal pain and other gastrointestinal symptoms by the time they visit the clinic, this is when the mitochondrial function of the pancreatic acini has almost completely collapsed. Previous studies by our group have identified MSCs as a potential clinical treatment option for SAP,^[^ ^8^, ^22^ ^]^ but due to the rapid course of SAP and the indications for MSC use, clinical trials have not been successfully conducted.

In recent years, numerous studies have demonstrated that MSCs can act as a “firefighter” in inflammatory injury, diverting mitochondria to target cells to alleviate inflammation.^[^ ^23^ ^]^ Our experiments confirm that hypoxic preconditioning not only upregulates mitochondrial function in MSCs, but also that mitochondria play a key role in this treatment. We suppose that transfer of hypoxic mitochondria alleviates further damage to PACs by upregulating glycolytic capacity, increasing glycolytic ATP production and driving ion pumps to maintain calcium homeostasis in PACs. Previous studies have indicated that the secretion or exclusion of mitochondria from MSCs under stressful conditions is not an “altruistic” act and is, rather a self‐protective mechanism to ensure their own survival by dislodging depolarized mitochondria.^[^ ^24^ ^]^ In addition, the relationship between mitochondria and glycolysis is still unclear. MitoDNA may be involved in the regulation of cellular glycolysis,^[^ ^25^ ^]^ but the exact mechanisms deserved further investigation. Undeniably, hypoxic pretreatment did enhance mitochondrial membrane potential levels and reduce mitochondrial superoxide accumulation in MSCs. Cargocytes detached from MSCs carried these functionally enhanced mitochondria to the injured pancreas to exert functionality.

In conclusion, our study successfully developed a highly effective and clinically transformative strategy for the treatment of SAP based on CFDV. We demonstrated that denucleated stem cells have an inflammatory chemotactic ability that is similar to MSCs to carry functional mitochondria and a variety of anti‐inflammatory factors; these cells can target sites of injury in the pancreas to exert therapeutic effects. Cargocytes donate mitochondria to injured PACs to maintain a relatively stable metabolic environment that provides adequate amounts of ATP for PACs to resist inflammatory stimuli. Because of the multiple advantages of Cargocytes, we anticipate that Cargocyte technology will open a new window for mitochondrial therapy and stem cell applications in the treatment of acute diseases.

## 4 Experimental Section

### Isolation, Culture, and Identification of Human Umbilical Cord‐Derived Mesenchymal Stem Cells (hUC‐MSCs)

Umbilical cords were obtained from cesarean sections performed on three healthy full‐term pregnant women after the acquisition of consent (and authorization approved by the Pudong New Area Gongli Hospital Ethics Review Committee: GLYYls2022‐028). MSCs were obtained from the umbilical cord using the tissue attach method, as previously described.^[^ ^13^ ^]^ When the cells had reached 80% fusion around the tissue, the cells were collected using 0.05% trypsin digestion and replanted at a density of 1 × 10^4^ cm^−2^, and the fetal bovine serum (FBS, 10099–141, Gibco, USA) in the medium was replaced with 5% platelet lysate (HPCPLCRL50, HELIOS, USA) to purify MSCs. The isolated P3 MSCs were stained for surface antigens using monoclonal antibodies and isotype controls to detect by flow cytometry. Stem cell surface markers (CD79, CD90, and CD105) were present on more than 95% of cells. Less than 1% of cells expressed CD31 and CD45, thus indicating that the isolated and expanded hUC‐MSCs were not contaminated with endothelial cells or hematopoietic cells (Figure S1A, Supporting Information). Differentiation experiments with the systemic medium also demonstrated the potential of the isolated hUC‐MSCs to differentiate into lipogenic (HUXUC‐90031, Cyagen, China), osteogenic (HUXUC‐90021, Cyagen, China) and chondrogenic lineages (HUXUC‐90041, Cyagen, China) (Figure S1B, Supporting Information). To ensure the stability of the cells, the P3–P6 generations of hUC‐MSCs were used in all experiments.

### Hypoxic Pre‐Treatment and Collection of Conditioned Media

Cells were cultured to 70% confluence in 75 cm^2^ flasks (#430641, Corning, USA), incubated with serum‐free *α*‐Minimum Essential Medium (*α*‐MEM, C12571500BT, Gibco, USA) for 30 min and washed twice with PBS to completely deplete residual platelet lysates. The medium was then removed and replaced with fresh serum‐free *α*‐MEM and maintained at 37 °C, 21% O_2_, 5% CO_2_ (normoxia) or 5% O_2_, 90% N_2_, 5% CO_2_ (hypoxia) for 48 h, respectively. The CM was then collected for EVs isolation.

### Isolation and Identification of MSC‐EVs

EVs were isolated from MSC conditioned medium using a Beckman Coulter ultracentrifuge (Optima XPN‐100), according to a previously described.^[^ ^9a^ ^]^ As shown in Figure S3A, Supporting Information: MSC‐CM was centrifuged at 300 × *g* and 2000 × *g* for 10 and 20 min at 4 °C to remove cell/cell debris and apoptotic vesicles, respectively. The supernatant was then transferred to an ultracentrifuge tube in a Ti‐400 bucket pendulum at 100 000 g for 2 h to obtain all the vesicles in the CM. Finally, the EV particles were resuspended in 200 µL ice‐cold PBS, and the protein concentrations of Norm‐EV and Hypo‐EV were checked using the Bicinchoninic acid protein assay (ZJ101, Epizyme Biomedical Technology, China). Western blotting was used to check for typical vesicle markers (Figure S3B, Supporting Information). The concentration and size of the EVs (Figure S3C, Supporting Information) were measured using ZetaVIEW (Particle Metrix, Germany) and the spherical bilayer structure of EVs (Figure S3D, Supporting Information) was observed using transmission electron microscopy (TEM). Proportion of positive mitochondria staining in isolated EVs using FACS flow cytometry.

### Isolation of Dysfunctional Mitochondria MSC‐EVs

To disrupt mitochondrial function, 1 µg mL^−1^ rhodamine‐6G (R4127, Merck, USA) was added to the MSC medium while supplementing with 50 µg mL^−1^ uridine (A610570, Sangon Biotech, China) and 275 µg mL^−1^ sodium pyruvate (A501259, Sangon Biotech, China) to support glycolysis, and treated for 48 h. Conditioned medium was collected and dysfunctional mitochondria EVs (Rho‐Norm‐EV and Rho‐Hypo‐EV) were isolated in the same way as normal MSC‐EVs.

### Isolation and Characterization of Rat Pancreatic Acinar Cells

Rats were euthanized and disinfected by immersion in 75% ethanol for 30 min; then, the abdomen was opened to block the hilar bile duct. Five milliliter of ice‐cold hypertonic citrate adenine solution was injected retrogradely through the opening of the pancreatic duct to completely fill the pancreatic lobules. Then, the vessels and fat were removed, the pancreatic lobes were cut into small pieces, and transferred the tissue to HBSS (14175095, Gibco, USA) that had been prewarmed to 37 °C containing 2 mg mL^−1^ of type I collagenase (1904MG100, Biofroxx, Germany) and 0.15 mg mL^−1^ of soybean trypsin inhibitor (SG2033, Beyotime, China). The tissue was digested at 37 °C for 25 min until the pancreatic tissue had been completely dissolved. The digestion solution was filtered through a 70 µm sieve and terminated with an equal volume of ice‐cold Dulbecco's Modified Eagle Medium (DMEM, 11995040, Gibco, USA) containing 10% FBS (04‐010‐1A, Biological Industries, China). Then, the supernatant was removed by centrifugation and the cell precipitate was resuspended in DMEM containing 10% FBS and 0.15 mg mL^−1^ of soybean protease inhibitor. The precipitate was incubated at 37 °C for 30 min for subsequent experiments. Isolated rPACs were identified by methylene blue staining (Figure S1C, Supporting Information).

### In Vitro Stimulation Experiments

To simulate the damaging effect of bile acids on rPACs, the cells were incubated for 4 h at 37 °C using serum‐free DMEM containing 4 mm sodium taurocholate (TCS, 86339, Sigma‐Aldrich, USA). Before adding TCS to stimulate the cells, 50 µg mL^−1^ of Norm‐EV, Hypo‐EV, Rho‐Norm‐EV, or Rho‐Hypo‐EV were added and incubated for 6 h respectively to complete EV internalization.

### Mitochondrial Labeling and Transfer

For the mitochondrial transfer study, MitoTracker fluorescent probes were used to label active mitochondria in cells. hUC‐MSCs were grown to 80% confluence, washed with PBS to remove any residual platelet lysate, before staining and pre‐staining with 200 nm of MitoTracker Red CMXRos (40741ES50, Yeasen, China) in 10 mL of serum‐free *α*‐MEM; this was then incubated in darkened conditions for 45 min. Then, cells were washed five times with PBS, replaced with fresh serum‐free *α*‐MEM, and MSC‐EVs were extracted, as described previously.

The mitochondria of rPACs were stained with 200 nm MitoTracker Green FM (40742ES50, Yeasen, China) for 45 min. Cells were twice with PBS to remove excess dye; then, the MSC‐EVs (50 µg mL^−1^) labeled with MitoTracker Red CMXRos were added and incubated for 6 h to allow EVs to internalized. After two washes with ice‐cold PBS, mitochondrial transfer was observed and photographed using an inverted confocal microscope (LSM780, Carl Zeiss, Germany) in the same *Z*‐axis plane (63×, oil lens).

### Mitochondrial Superoxide (Mito‐SOX) Assay

Mito‐SOX production was measured by flow cytometry according to the instructions of the Mito‐SOX Red (40778ES50, Yeasen, China) kit. hUC‐MSCs were collected by centrifugation after 0.05% trypsin digestion, and the cells were resuspended using a pre‐configured MitoSOX staining working solution (2.0 mL, 5 µm) for 10 min at 37 °C. Excess dye was then removed by washing three times with PBS. The average fluorescence intensity of the cells was measured by flow cytometry (*λ*_Ex_/*λ*_Em_: 510/580 nm).

### Apoptosis and Necrosis Assay

Treated rPACs were stained with propidium iodide and Hochest33342 according to the instructions of the Necrosis and Apoptosis Staining Kit (C1056, Beyotime, China). Then, the fluorescence intensity of the PI and DAPI channels was detected by flow cytometry. Data analysis was performed using FlowJo software (FlowJo X10.0, USA).

### Detection of Mitochondrial Membrane Potential

rPACs mitochondria were labeled using 50 nm tetramethylrhodamine methyl ester (TMRM, T17109L, Topscience, China) for 20 min after loading with different EVs. Changes of mitochondrial membrane potential were observed continuously (*λ*_Ex_/*λ*_Em_: 530/592 nm, photographed at 5 s intervals) using an LSM 780 system within 15 min of stimulation with 4 mm TCS.

### Mitochondrial Isolation from hUC‐MSCs

Mitochondria were isolated from hUC‐MSCs according to the protocol recommended in the Beyotime Cell Mitochondrial Isolation (C3601, Beyotime, China) kit and the mitochondria was resuspended in ice‐cold mitochondrial respiration buffer (C3609, Beyotime, China). Isolated mitochondria were used for experiments within 30 min.

### Preparation and Characterization of Cargocytes

hUC‐MSCs were enucleated by ultracentrifugation using a density gradient of Ficoll PM400 (ST1321, Beyotime, China) and cytochalasin B (MB5434, Meilunbio, China) according to previously described methods.^[^ ^14^ ^]^ Ficoll PM400 was dissolved into 50% w/w ultrapure water with continuous magnetic stirring at RT until completely dissolved, then autoclaved. 50% Ficoll PM400 solution was diluted to 25% with 2X MEM (11935046, Gibco, USA) and then further diluted to 20%, 17%, 16%, 15%, and 12.5% Ficoll solutions with *α*‐MEM. Cytochalasin B was added to all Ficoll solutions at a final concentration of 10 µg mL^−1^. 2 mL of 25%, 1 mL of 20%, 1 mL of 17%, 1 mL of 16%, 2 mL of 15% and 2 mL of 12.5% density Ficoll solution were added to the ultracentrifuge tubes in that order and incubated overnight at 37°C. Then, hUC‐MSCs were harvested, resuspended in a 12.5% Ficoll solution, and added to the top layer of the continuous gradient of Ficoll solution. Ultracentrifuge tubes were balanced, placed in a horizontal swing rotor, and centrifuged at 100 000 × *g* for 1 h at 35 °C. The minimum centrifuge braking parameters were set and the cells were recovered from the middle layer of the Ficoll solution using a low‐binding tip at the end of centrifugation. Then, the cells were washed five times with serum‐free *α*‐MEM and cryopreservation CELLSAVING medium (C40100, New Cell&Molecular Biotech, China) at −80 °C or used immediately for subsequent experiments. Next, the number and diameter of Cargocytes in a Cellometer (AUTO T4, Nexcelom, USA) were measured. Mitochondria, cell membranes, and the nuclei of Cargocytes were stained using 200 nm MitoTracker Red CMXRos at 37 °C for 45 min, 3 µm DiO (40725ES10, Yeasen, China) for 20 min and 1 µg mL^−1^ Hochest33342 (C1022, Beyotime, China) for 10 min and then observed by confocal microscopy.

### Transwell Migration Assay

5 × 10^4^ of hUC‐MSC or Cargocytes were added to the upper chamber of the Transwell device (3422, Costar, USA). After 8 h of incubation, the upper chamber was fixed with 4% paraformaldehyde for 15 min, stained with 0.1% crystal violet for 15 min in the dark, and photographed with a phase‐contrast microscope.

### Animal Experimental SAP and Follow‐Up Treatment

All animal experiments are approved by the Institutional Animal Care and Use Committee (IACUC) of Tongji University School of Medicine. The animal welfare of the experimental rats was in accordance with the Guide for the Ethical Review of Laboratory Animals‐Animal Welfare (GB/T 35892‐2018). Male SD/SPF rats (6 weeks‐of‐age) were provided by Charles River Laboratory (Zhejiang, China) and were acclimatized at the Animal Centre of the Shanghai Tenth People's Hospital for 7 days prior to the experiment and fasted for 12–16 h prior to surgery. The model of SAP was induced as previously described.^[^ ^15^ ^]^ MSCs/Cargocytes (1 × 10^6^ kg^−1^), MSC‐EVs (1 mg kg^−1^), and mitochondria (isolated from 1 × 10^6^ cells) were injected via the tail vein at 6 h following the induction of TCS. The control group was injected with an equal volume of solvent vehicle. Then, 72 h after the induction of SAP, rats were euthanized and pancreatic tissue, peripheral blood and other organs were harvested for subsequent experiments.

### In Vivo Tracing of hUC‐MSCs and Cargocytes

1,1‐dioctadecyl‐3,3,3,3‐tetramethylindotricarbocyaine iodide (DiR, 40757ES25, Yeasen, China) was selected as an in vivo tracer. Cells were incubated with 4 µm DiR staining solution at 37 °C for 10 min and then washed three times with PBS to remove excess dye to obtain DiR‐labelled cells. The rats were photographed using a multimodal animal live imaging system (AniView100, China). AniView (v1.00.0045, China) was used to analyze the bioluminescence signal intensity in the region of interest (ROI); this way divided by the area of the region as the mean fluorescence intensity (MFI) for each animal.

### Histopathology

Rat pancreatic tissue was trimmed and fixed in 4% paraformaldehyde for 48 h, gradient dehydrated, and embedded in paraffin for sectioning. Slices were stained with hematoxylin and eosin (H&E) and two experienced pathologists scored the degree of pancreatic damage according to a previous scale in a double‐blind manner (Table S1, Supporting Information).

### Detection of Biochemical Indicators in Serum and Tissues

Trypsin activity (A080‐2‐2, Nanjing Jiancheng Bioengineering Institute, China) in pancreatic tissue and the serum levels of amylase (C016‐1‐1, Nanjing Jiancheng Bioengineering Institute, China) and lipase (A054‐2‐1, Nanjing Jiancheng Bioengineering Institute, China) were analyzed using commercial kits in accordance with the manufacturer's instructions.

### ELISA

The serum levels of IL‐1*β* (EK301B, MULTI SCIENCES, China), IL‐6 (EK306, MULTI SCIENCES, China), and IL‐10 (EK310, MULTI SCIENCES, China) were measured using ELISA kits according to the manufacturer's protocol.

### ATP Assays

ATP production in pancreatic tissue or rPACs was measured with an enhanced ATP assay kit (S0027, Beyotime, China) according to the manufacturer's protocol.

### Transmission Electron Microscopy

*EVs*: MSC‐EVs were fixed using 500 µL of 4% paraformaldehyde. Then, the EVs were resuspended in 30 µL of PBS and then 10 µL of each sample was placed on formvar/carbon‐coated grids, dried at room temperature, and then fixed with 2% glutaraldehyde for 10 min. Next, 1% tannic acid ACS reagent (Sigma‐Aldrich, UK) and TAAB EM Heavy Metal Stain 336 (TAAB, Laboratory Equipment Ltd., UK) were used for staining. Finally, MSC‐EVs were visualized using a TEM microscope (HT7800, HITACHI, Japan).

*Cells*: 1 × 10^6^ cells were fixed in 2.5% glutaraldehyde (pH 7.4) at 4 °C for 24 h. Cells were precipitated by embedding in 1% agarose and then fixed in 1% osmium acid for 2 h and dehydrated in a gradient series of acetone and ethanol. Next, ultra‐thin sections of 60 nm thickness were prepared using an ultra‐thin sectioning machine (UC7, Leica, America). These sections were then stained with 2% dioxygen acetate and 2.6% lead citrate. Finally, the sections were observed by TEM microscopy.

### Immunohistochemical Staining

Paraffin‐embedded tissue samples were cut into 4 µm‐thick sections, which were dewaxed and dehydrated. Following antigen repair, the sections were blocked with goat serum for 1 h and incubated with antibodies against MPO (1:500 dilution, AF7494, Beyotime, China), followed by a goat‐anti‐rabbit secondary antibody (G1213, Servicebio, China). Subsequently, the tissue sections were stained with 3,3′‐diaminobenzidine and hematoxylin. Images were acquired by an optical microscopy.

### Immunofluorescence Assay

Cells or tissue sections were permeabilized with 0.1% Triton X‐100 for 10 min, blocked with goat serum for 30 min, and incubated with HMGB1 (1:100 dilution, T55060, Abmart, China), TOMM20 (1:100 dilution, CL594‐66777, Proteintech, China), or F‐actin (1:200 dilution, C2201S, Beyotime, China) antibodies for 16–18 h at 4 °C. The next morning, the sections were incubated for 1 h at room temperature with ATF594‐coupled secondary antibody (direct label antibodies do not require secondary antibody staining). Cell nuclei were re‐stained with DAPI. Fluorescence images were acquired by a confocal fluorescence microscopy.

### Western Blotting

Pancreatic tissue, cells, or EV pellets were lysed in radioimmunoprecipitation assay lysis buffer (RIPA, PC101, Epizyme, China) containing 1% Protease Inhibitor Mix (GRF101, Epizyme, China) and the protein concentration obtained after centrifugation was measured using the BCA Protein Assay Kit (ZJ101, Epizyme, China). The same amount of each protein sample (30 µg/lane) was separated by electrophoresis and transferred to nitrocellulose filter membranes. The membranes were then blocked with 5% milk in TBST for 1 h at room temperature and then incubated with primary antibody (Table S2, Supporting Information) overnight at 4 °C. Then, the membranes were incubated with the secondary antibody for 1 h at room temperature. Immunoreactive protein bands were visualized with an enhanced chemiluminescence system (Amersham Imager 600; GE Healthcare Bio‐Sciences Corp., USA) or an Odyssey M Infrared Imaging System (LI‐COR Biosciences, USA) and the bands were analyzed for grey scale values using ImageJ software (National Institutes of Health, USA).

### Seahorse Analysis

The bioenergetic profile of PACs loaded with different EVs after TCS treatment was assessed using XF96 analyzer (Seahorse Bioscience, Agilent) following the recommended protocol of the reagent. Mitochondrial respiration and glycolysis were reflected by real‐time measurements of oxygen consumption rate (OCR) and extracellular acidification rate (ECAR).

The day before the experiment, probe plates were hydrated using autoclaved double‐distilled water overnight at 37 °C in a CO_2_‐free incubator; rat PACs were extracted, mixed with isolated EVs, and incubated overnight at 37 °C to allow the EVs to internalized.

On the day of the experiment, the PACs were stimulated with 4 mm TCS for 30 min, then the cells were resuspended using XF assay medium (103576‐100, Agilent, USA) and 180 µL medium (≈5 × 10^4^ cells) was slowly added to each well of cell plates to avoid bubbling. Cell plates incubated for 1 h at 37 °C, CO_2_‐free incubator to expel CO_2_. The probe plates were calibrated using XF calibration solution and incubated for 1 h at 37 °C, CO_2_‐free incubator. During incubation, inhibitors were added to the side wells of the probe plate cover. The probe plate and cell plate were placed in the analyzer and the OCR or ECAR was measured according to the pre‐set parameters. The original data was analyzed using Wave software (version 2.6, Agilent) according to the manufacturer's instructions.

### RNA Sequencing

The preparation and sequencing of the RNA‐seq libraries were performed by Shanghai Genechem Co. In brief, total RNA was extracted from hUC‐MSCs using TRIzol reagent, cDNA libraries for RNA‐seq analysis were then prepared using an Illumina NEBNext UltraTM RNA Library Prep Kit. Universal bridging substrates were ligated to cDNA fragments and the sequencing libraries were tested for quality with an Agilent 2100 Bioanalyzer (Agilent, Santa Clara, USA). The constructed cDNA libraries were sequenced on the HiSeq 2000 platform (TruSeq SBS KIT‐HS V3, Illumina) with paired ends sequenced to 150 bp. Gene expression levels were determined and differentially expressed genes (DEGs) were identified using the method described by Audic and Claverie.^[^ ^16^ ^]^ In the case of multiple transcripts of a gene being detected, the longest reads were used to calculate their expression levels and coverage. Significant DEGs were identified at false discovery rate thresholds ≤ 0.001 and absolute log 2 ratio values ≥ 1.0. using the “limma,” “corrplot,” and “clusterProfiler” packages in R (v4.1.1, New Zealand) to generate DEG heat maps, correlation maps, and allow enrichment analysis.

### Preparation of Samples and Data Analysis for Metabolomics Analysis

Following euthanasia, the aortic arch of each rat was cannulated and the right auricular was cut. Then, 250 mL of ice‐cold normal saline was instilled to remove the blood until clear fluid flowed from the right heart ear. The pancreas was removed and placed in liquid nitrogen for rapid quenching. Then, 100 mg of tissue was ground in liquid nitrogen and transferred to a microcentrifuge tube with 500 µL of cold methanol (Sigma‐Aldrich, Cat# 646377)/water (8:2), vortexed and shaken, left to stand in an ice bath for 5 min, and centrifuged at 15 000 × *g* for 20 min at 4 °C. Next, 300 µL of supernatant was diluted with mass spectrometry grade water to 53% methanol, centrifuged at 15 000 × *g* for 20 min at 4 °C, and supernatant was collected for analysis by Ultra‐High Performance Liquid Chromatography Coupled to Mass Spectrometry (UHPLC/MS). The UHPLC‐MS/MS assay was performed with a UHPLC system (Vanquish, Thermo Fisher Scientific) equipped with a Waters Acquity BEH C18 column (100 × 2.1 mm, 1.9 µm, Waters). MS detection was determined by a Q Exactive HFX mass spectrometer (Thermo Fisher Scientific) in parallel reaction monitoring (PRM) mode. Raw data (.raw) files were imported into Compound Discoverer (CD 3.1) software for processing.

To further characterize the metabolic changes and pathways involved, individual metabolites were processed and analyzed using MetaboAnalyst 5.0 (www.metaboanalyst.ca). Pathway analysis and enrichment analysis were based on the KEGG pathway database and the small molecule pathway database (SMPDB).

### Statistical Analysis

Data analysis was performed using GraphPad Prism (V 9.0, San Diego, CA). Data are expressed as the mean ± standard deviation (SD). For statistical analysis of data obtained from *n* ≥ 5 independent experiments, parametric tests were used after the validation of normality and equality of variances of populations by using Shapiro–Wilks and Browne–Forsythe tests, respectively. Otherwise, non‐parametric tests, including one‐way ANOVA on rank followed by Dunn's multiple comparisons were used. For statistical analysis of data obtained from *n* < 5 experiments, non‐parametric tests were applied. In particular, metabolomics data were analyzed by one‐way ANOVA on rank followed by Conover‐Iman pairwise comparisons and the *p*‐values were adjusted with the Benjamini–Hochberg method to control the false‐discovery rate (FDR).^[^ ^17^ ^]^ Results were considered significant when *p* < 0.05.

### Ethics Approval Statement

Umbilical cord tissue acquisition was authorized by the approval of the Ethics Review Committee of the Pudong New Area Gongli Hospital (NO. GLYYls2022‐028).

## Conflict of Interest

The authors declare no conflict of interest.

## Supporting information

Supporting Information

## Data Availability

The data that support the findings of this study are available in the supplementary material of this article.
