# Supplementary material for: MSCs Deliver Hypoxia‐Treated Mitochondria Reprogramming Acinar Metabolism to Alleviate Severe Acute Pancreatitis Injury
Source: Adv Sci (Weinh). 2023 Jul 6;10(25):2207691. doi: 10.1002/advs.202207691 (PMC10477874; doi:10.1002/advs.202207691)
Supplement: Supplementary file 1 — Supporting Information [file ADVS-10-2207691-s001.pdf]

ADVANCED SCIENCE
Open Access

Supporting Information

for *Adv. Sci.*, DOI 10.1002/advs.202207691

MSCs Deliver Hypoxia-Treated Mitochondria Reprogramming Acinar Metabolism to Alleviate Severe Acute Pancreatitis Injury

Zhengyu Hu, Dongyan Wang, Jian Gong, Yan Li, Zhilong Ma, Tingyi Luo, Xuyang Jia, Yihai Shi and Zhenshun Song**

Cargocytes deliver hypoxia-treated mitochondria reprogramming acinar metabolism to alleviate severe acute pancreatitis injury

Zhengyu Hu[#], Dongyan Wang[#], Jian Gong[#], Yan Li, Zhilong Ma, Tingyi Luo, Xuyang Jia, Yihai Shi[*], Zhenshun Song[*]

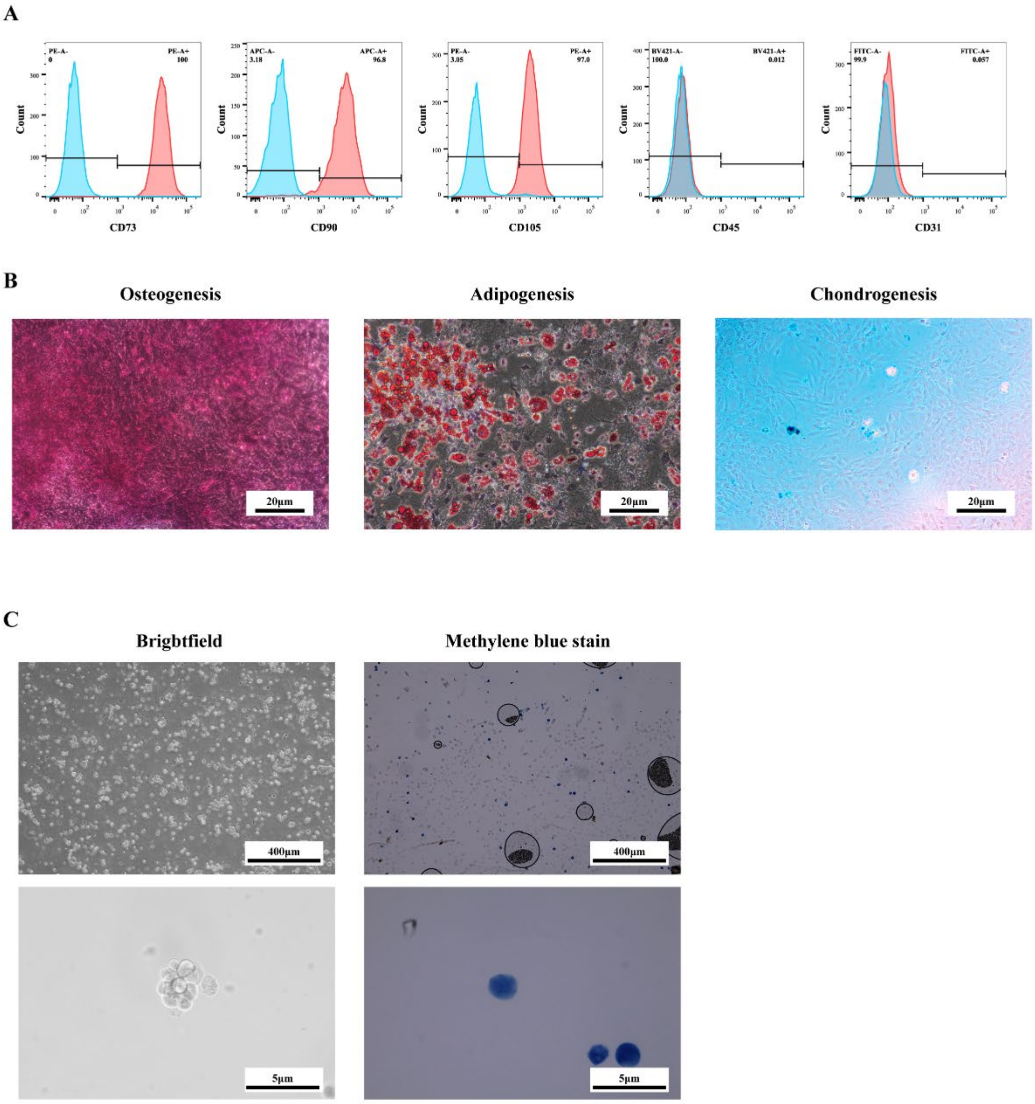

Figure S1. Identification of primary cells. A) Flow cytometry analysis of P3 Generation UC-MSCs. Red ridges represent isotype controls. B) Representative images of osteogenic (left panel), lipogenic (middle panel) and chondrogenic (right panel) differentiation of UC-MSCs. C) Representative images of bright field and methylene blue staining of primary rPACs.

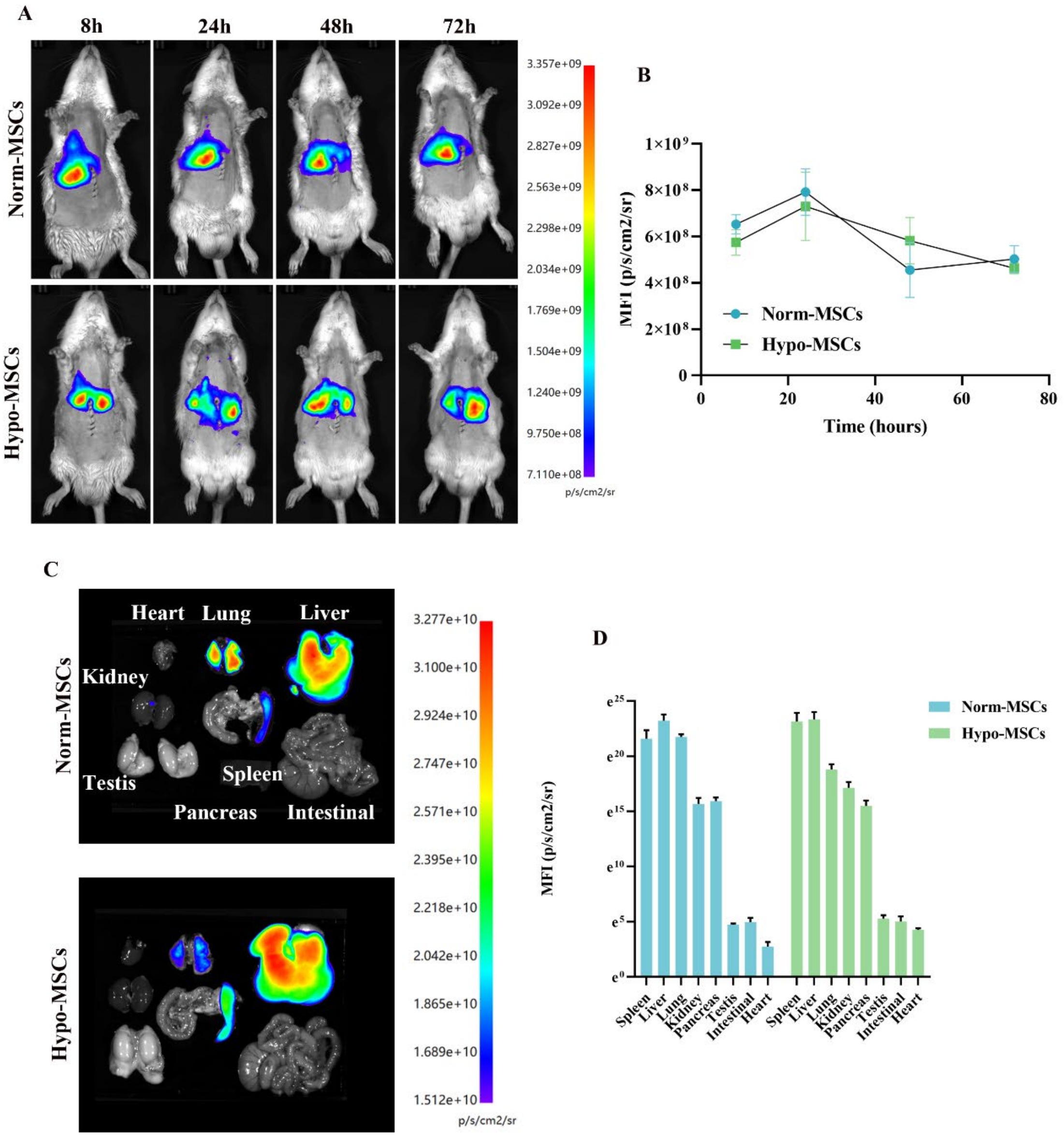

Figure S2. Tracking the distribution of hUC-MSCs injected into rats via the tail vein. A-B) The results of in vivo imaging show a gradual accumulation of DiR-labelled hMSCs in the liver and spleen (A). Quantitative analysis of fluorescence intensity showed a peak at 24 hours post-injection, followed by a gradual decrease (B). C, D) Detection and quantification of autofluorescence in heart, lung, liver, kidney, pancreas, spleen, testis and intestinal ducts of rats.

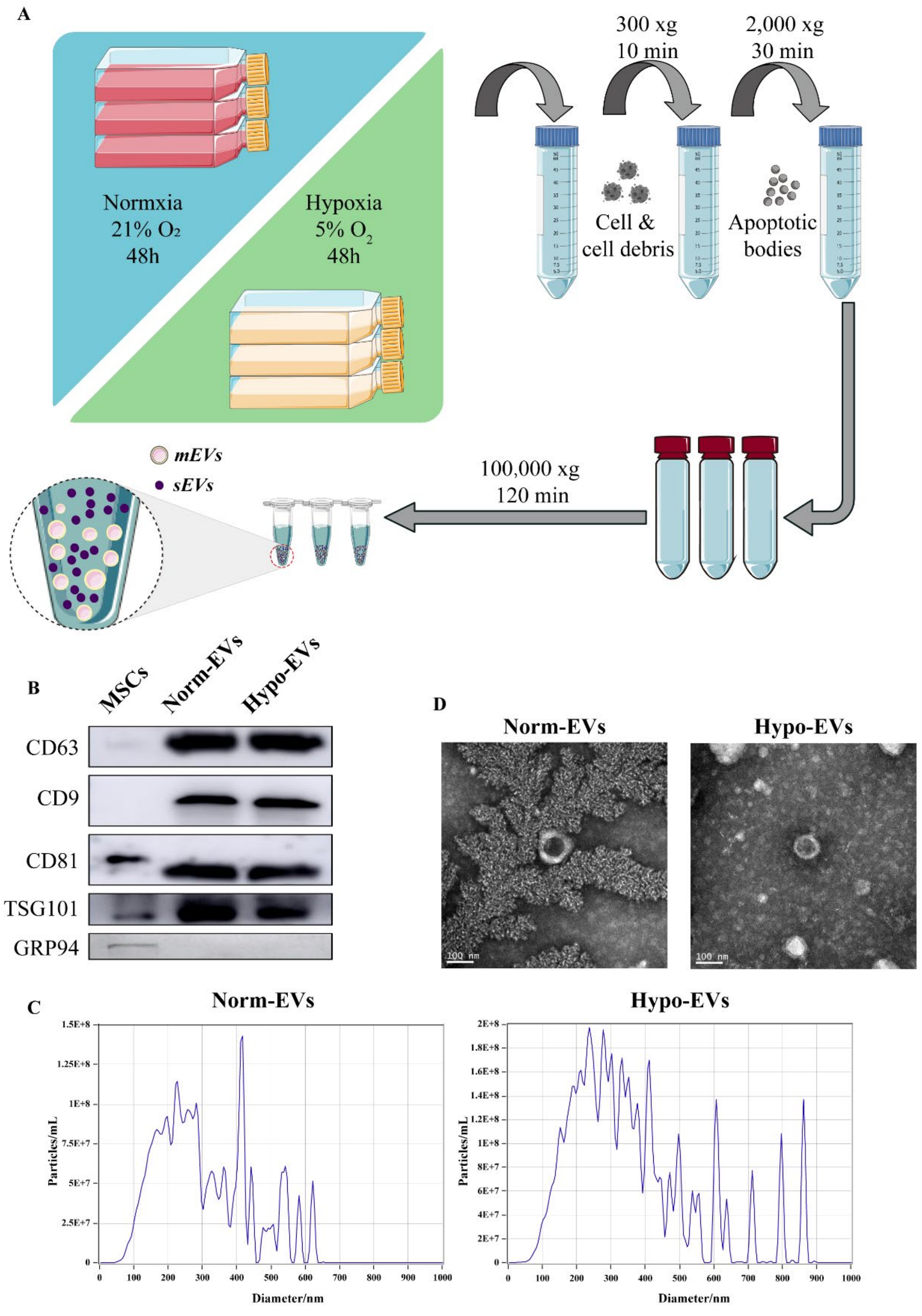

Figure S3. Separation and characterization of hUC-MSC-derived EVs. A) Schematic depicting the workflow used to separate the conditioned medium (CM) and the different secretome fractions. B) Western blotting analysis of EVs' markers. C) Representative histograms showing the size distribution by NTA analysis of purified EVs. D) Representative TEM images of separated EVs.

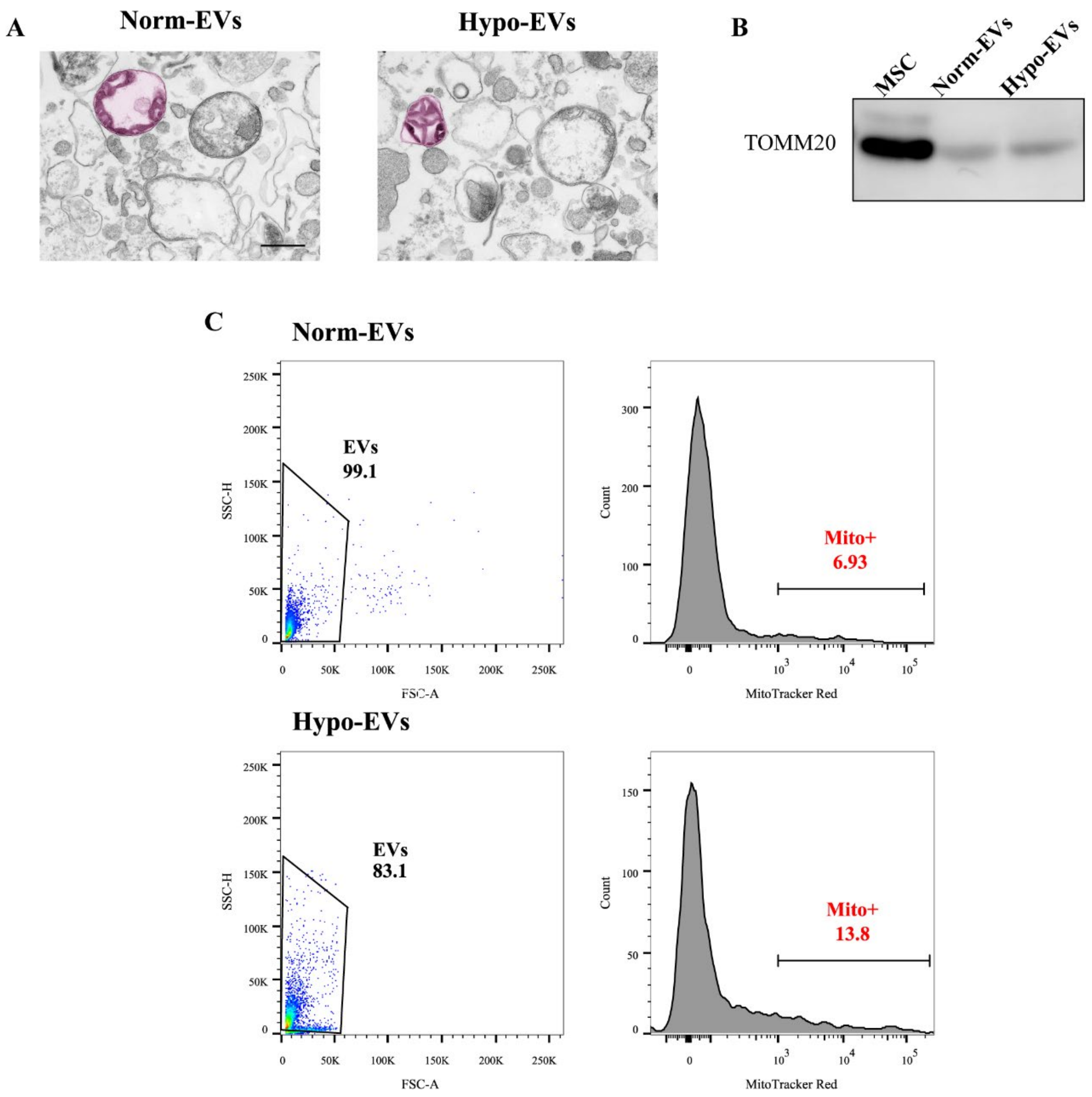

Figure S4. Characterization of mitochondria in MSC-EVs. A) Representative transmission electron microscopy (TEM) images showing: EVs contain mitochondria like structures, scale bar: 500 nm. B) WB detected mitochondrial marker TOM20 expression in Norm-EVs and Hypo-EVs lysates. C) The isolated EVs and exosomal mitochondria were characterized using FACS flow cytometry.

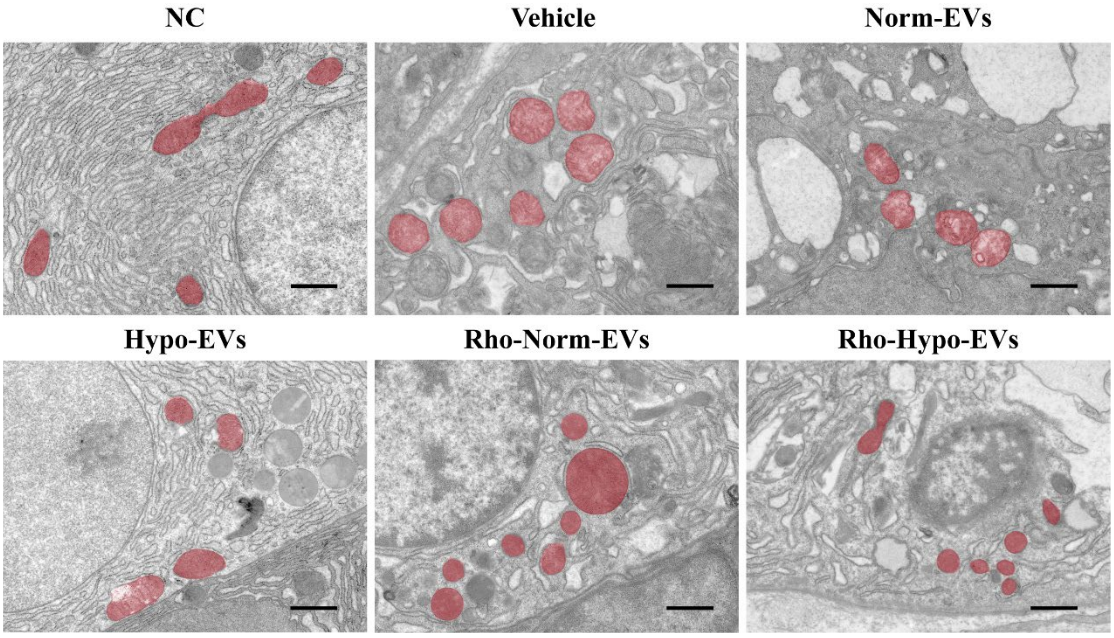

Figure S5. Representative TEM images of mitochondria in normal control and differently treated PACs, Scale bar, 1μm.

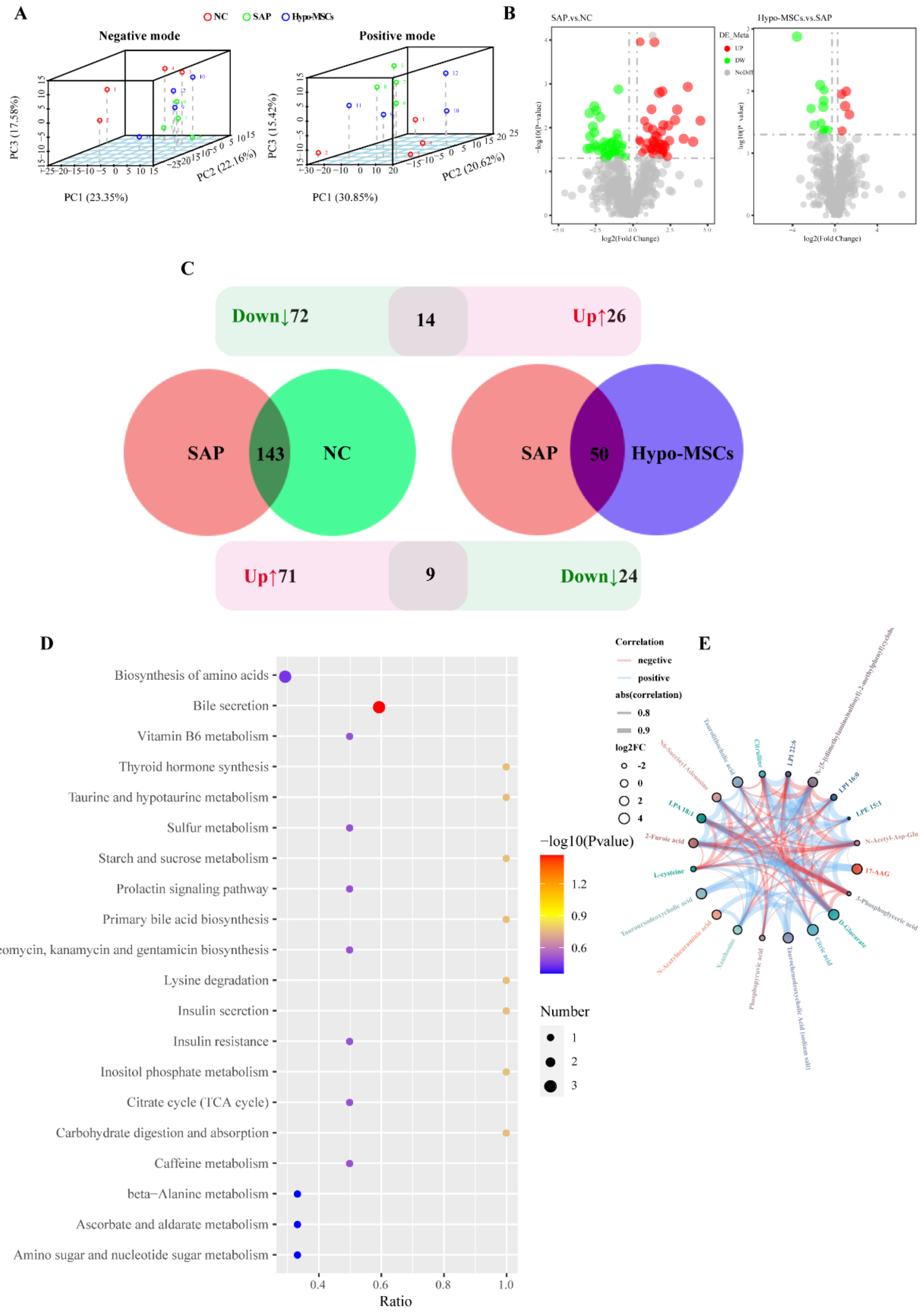

Figure S6. Reversal of mitochondrial function drives metabolic reprogramming of pancreatic tissue. Related to Figure 6. A) Samples are separated in the principal component analysis (PCA) with PC1, PC2, and PC3. B) Volcano map showing differential metabolites between NC and SAP (left panel), Hypo-MSCs and SAP (right panel). C) Venn diagram showing reversed metabolism. D) Enrichment analysis based on the SMPDB of reversal metabolites in the pancreas after Cargocytes treatment. E) Correlation between differential metabolites.

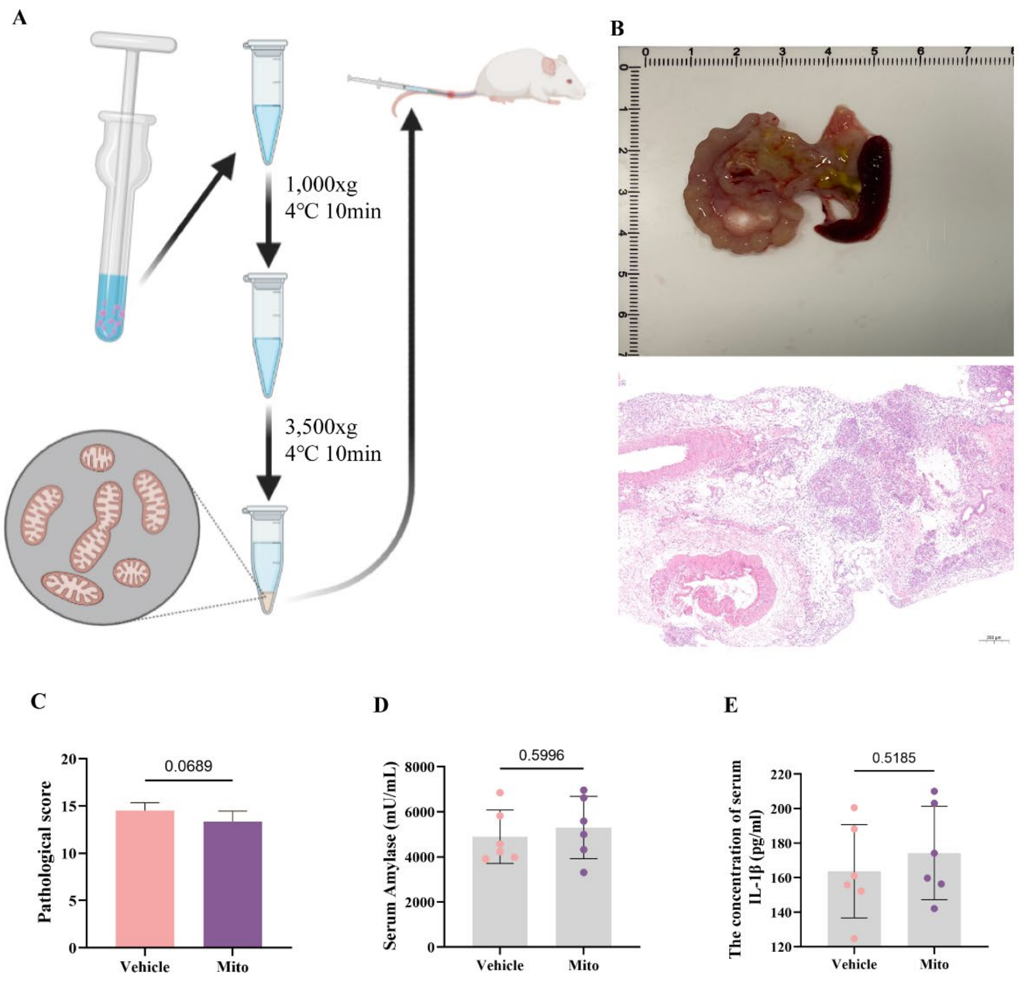

Figure S7. Purified mitochondria for SAP treatment. A) Schematic depicting the workflow used to separate the mitochondria. B) Representative images of pancreatic tissue and HE staining after mitochondrial treatment for SAP. C-E) Comparison of pathology scores (C), serum amylase (D) and IL-1β (E) between mitochondrial treatment and vehicle control group.

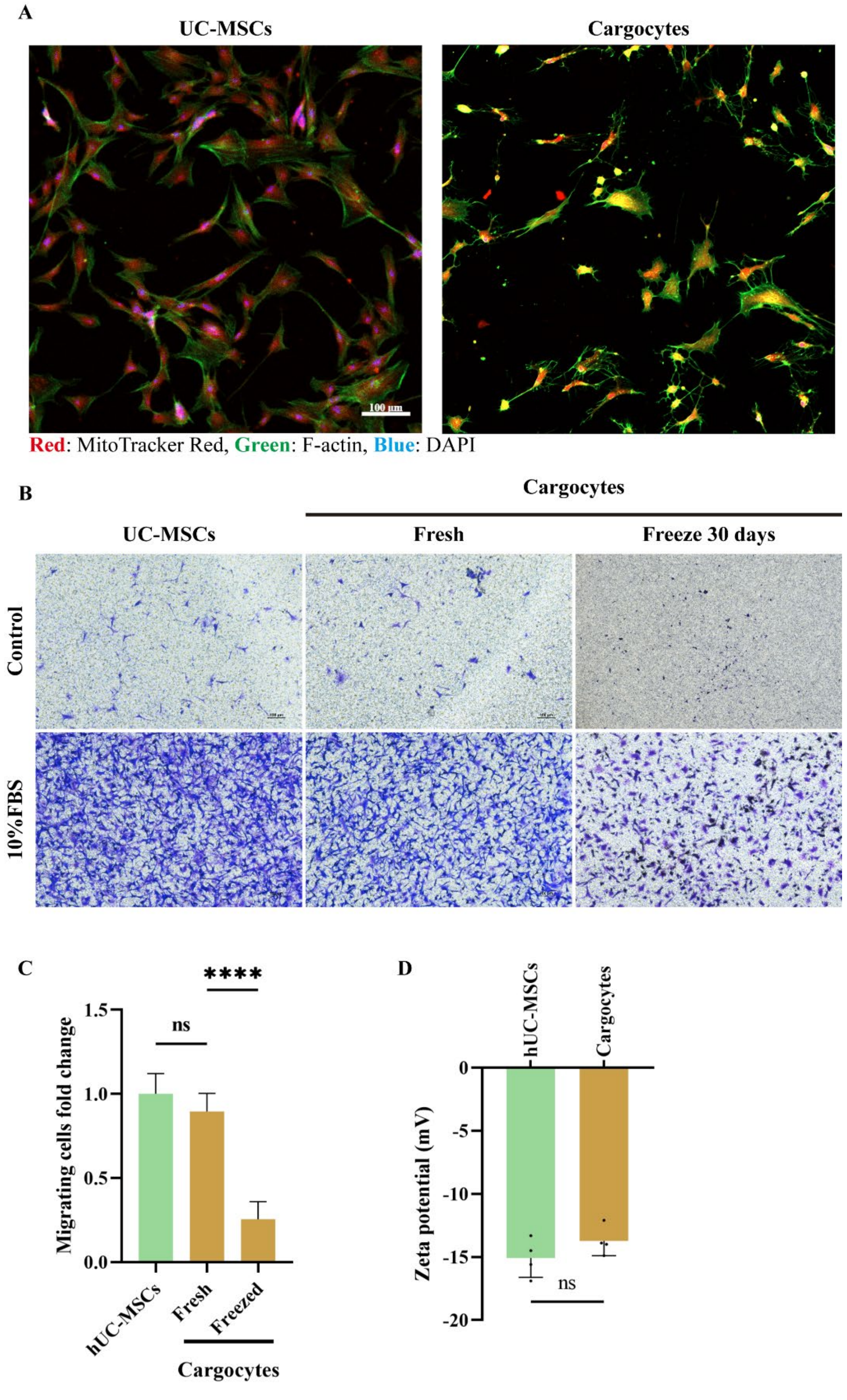

Figure S8. Cargocytes in vitro functional assay. A) Adherent state of Cargocytes (right panel) compared to UC-MSCs (left panel). Scale bar, 100μm. B) MSCs/Cargocytes migrated in Transwell chambers towards FBS (Fetal bovine serum) gradients for 2hr. Representative brightfield images of MSCs or Cargocytes that migrated to the underside of 8.0μm porous filters were stained with Crystal Violet. Scale bar, 100μm. C) Bar graphs represent the fold change of the migrating cells number (relative to UC-MSCs). D) Bar graph shows the zeta potential of EVs from MSCs or Cargocytes.

Table S1. Histological scoring for SAP.

Scores	**Edema**	**Inflammatory cellular infiltration**	**Vacuolization**	**Necrosis**
0	absent	absent	absent	absent
1	diffuse expansion of interlobar septa	around ductal margin	periductal, <5%	1–4 necrotic cells/HPF [a]
2	diffuse expansion of interlobular septa	in parenchyma, < 50% of lobules;	focal, 5–20%	5–10 necrotic cells/HPF
3	diffuse expansion of interacinar septa	in parenchyma, 50–75% of lobules	diffuse, 21–0%	11–15 necrotic cells/HPF
4	diffuse expansion of intercellular septa	in parenchyma, >75% of lobules	severe, >50%	≥16 necrotic cells/HPF

[a] HPF: high-power field.

Table S2. Antibody details

Name	Cat No.	Company	Dilutions
Primary antibodies			
β-Actin	P30002	Abmart	1:10000
NFκB (p65)	#8242	CST	1:1000
p-NFκB (p-p65)	#3036	CST	1:1000
CD63	M051014	Abmart	1:1000
CD9	T55337	Abmart	1:1000
CD81	T55742	Abmart	1:1000
TSG101	T55985	Abmart	1:1000
GRP94	60012-2-Ig	Proteintech	1:1000
TOMM20	11802-1-AP	Proteintech	1:1000
P62	#39749	CST	1:1000
LCIII A/B	#4108	CST	1:1000
CHOP	#2895	CST	1:1000
XBP1s	#40435	CST	1:1000
HIF-1α	NB100-105	NOVUS	1:1000
BNIP3	#44060	CST	1:1000
BNIP3L/NIX	#12396	CST	1:1000
OXPHOS cocktail	45-8099	Invitrogen	1:1000
PKM1/2	#3190	CST	1:1000
p-PKM	#3827	CST	1:1000
HK2	#	CST	1:1000
MPO	AF7494	Beyotime	1:500
HMGB1	T55060	Abmart	1:100
TOMM20	CL594-66777	Proteintech	1:100
Amylase	66133-1-Ig	Proteintech	1:100
DAPI	C1002	Beyotime	1:1000
Secondary-antibodies			
800CW Goat anti-Rabbit IgG	926-32211	LI-COR	1:1000
800CW Goat anti-Mouse IgG	926-32210	LI-COR	1:1000
HRP-conjugated Affinipure Goat Anti-Rabbit IgG	SA00001-2	Proteintech	1:1000
HRP-conjugated Affinipure Goat Anti-Mouse IgG	SA00001-1	Proteintech	1:1000
Goat Anti-Rabbit lgG AF 594	M21014	Abmart	1:1000
Goat Anti-Mouse lgG AF 488	M21011	Abmart	1:1000
